# Averting a public health crisis in England’s coastal communities: a call for public health research and policy

**DOI:** 10.1093/pubmed/fdab130

**Published:** 2021-05-13

**Authors:** Sheena Asthana, Alex Gibson

**Affiliations:** Plymouth Institute of Health and Care Research (PIHR), University of Plymouth, Drake Circus, Plymouth PL4 8AA, UK; Plymouth Institute of Health and Care Research (PIHR), University of Plymouth, Drake Circus, Plymouth PL4 8AA, UK

**Keywords:** children and young people, coastal health, communities, deprivation, geography, Quality and Outcomes Framework, small area mapping, social determinants

## Abstract

Coastal communities have received little attention in the public health literature, perhaps because our mental maps tend to associate socio-economic deprivation and health inequalities with inner cities. Mapping a range of key health indicators at small area level, this paper reveals a distinct core-periphery pattern in disease prevalence, with coastal communities experiencing a high burden of ill health across almost all conditions included in the Quality and Outcomes Framework dataset. Other sources suggest poor outcomes for children and young people living in coastal areas. Low rates of participation in higher education contrast with high rates of hospitalisation for self-harm, alcohol and substance use. Reflecting a shift in the distribution of children living in poverty since the 1990s, this may be an early indicator of a future public health crisis in these communities. Exploring reasons for the health challenges facing the periphery, this perspective piece calls for more public health research that can accommodate the complex and interlinked problems facing coastal communities and a more concerted effort to align public health with economic, education, local government and transport policies at the national level.

## Introduction

Just as inner-city deprivation was ‘discovered’ in the 1970s, the many challenges facing coastal communities in modern Britain are in urgent need of recognition. Notwithstanding a profound shift in deprivation (particularly of children living in poverty) away from cities and towards coastal areas,^1^ national policies to ‘level up’ opportunities and outcomes appears to be framed in terms of north–south rather than core-periphery inequalities. Economic (including infrastructural) investment continues to be strongly targeted at London and other large cities under initiatives such as the ‘Northern Powerhouse’. Per capita levels of social expenditure are, with the exception of NHS allocations, lower in deprived coastal areas than in their non-coastal equivalents. Educational expenditure is particularly highly skewed towards the best performing region, London.

This lack of policy attention may reflect the fact that, with the exception of seaside resorts,^2–6^ there has been limited research on the problems experienced by coastal communities in different parts of the country. The lack of an official definition of coastal communities and the poor granularity of most publicly available data has undoubtedly hindered research. The needs of coastal communities may also have been overlooked because of perceptions of the coast as part of ‘rural idyll’^7^; a place where economic opportunities are sacrificed by those who choose to pursue a coastal life.^8^ There has certainly been recent public health interest in the positive effects of coastal proximity on health and well-being,^9–11^ although the extent to which this can mitigate against the effects of lower than average wages, seasonal jobs, low skills, poor education attainment and social immobility is debatable.

The aim of this perspective piece is to demonstrate how poor health and public health outcomes are now subject to a significantly peripheral distribution in England. To this end, we have drawn on a range of data to map a series of key health indicators at Lower and Middle Layer Super Output Area (LSOA/MSOA) level. We explore reasons for the health challenges facing the periphery. Given mapped evidence of particularly worrying trends among children and young people, we highlight the role of education as an important—and modifiable—health determinant. In the final section of the paper, we call for more public health research on the complex and interlinked problems facing coastal communities and a more concerted effort to align economic, education and public health policies at the national level.

## Mapping coastal health: evidence of a future public health crisis on the periphery?

### Non-communicable diseases

**Fig. 1:**
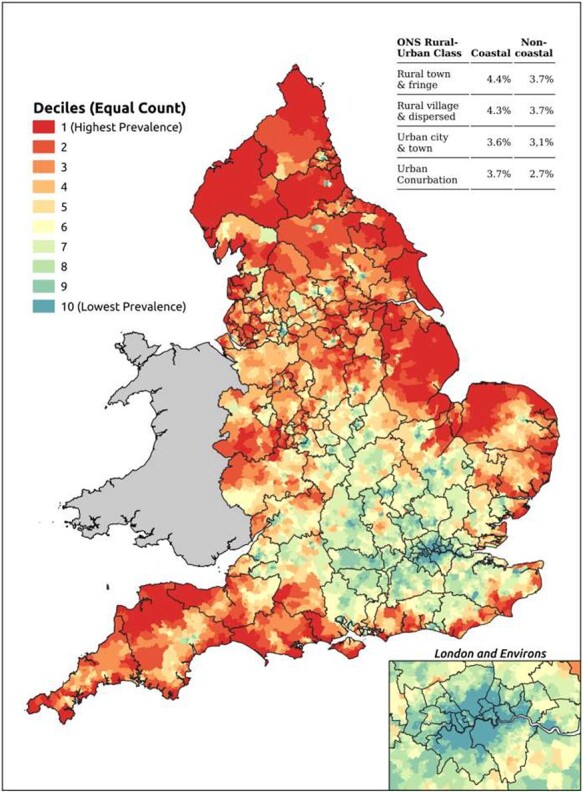
Coronary heart disease, QOF prevalence (LSOAs), 2014/15–2018/19. All maps based on digital boundaries obtained via the ONS Open Geography Portal (https://geoportal.statistics.gov.uk/). Source: Office for National Statistics licensed under the Open Government Licence v.3.0. Contains OS data © Crown copyright and database right [2021]. Specifically: *Lower Layer Super Output Areas (December 2011) Boundaries Generalised Clipped (BGC) EW V3.* [Online. Accessed 20/02/2021] (https://geoportal.statistics.gov.uk/datasets/lower-layer-super-output-areas-december-2011-boundaries-generalised-clipped-bgc-ew-v3); *Clinical Commissioning Groups (April 2020) EN BFC V2*. [Online. Accessed 20/02/2021] (https://geoportal.statistics.gov.uk/datasets/clinical-commissioning-groups-april-2020-en-bfc-v2); *Countries (December 2011) Boundaries EW BGC.* [Online. Accessed 20/02/2021] (https://geoportal.statistics.gov.uk/datasets/countries-december-2011-boundaries-ew-bgc).

The scale of the problem already faced by coastal communities is graphically illustrated by mapping the crude prevalence of coronary heath disease (Fig. 1). A core region of almost universally low rates focused on London and adjacent counties is surrounded by higher rates across many northern and western areas and around much of the coastal periphery. Other cardiovascular conditions for which quality outcomes framework (QOF) disease register data are available show a similar pattern.

**Fig. 2:**
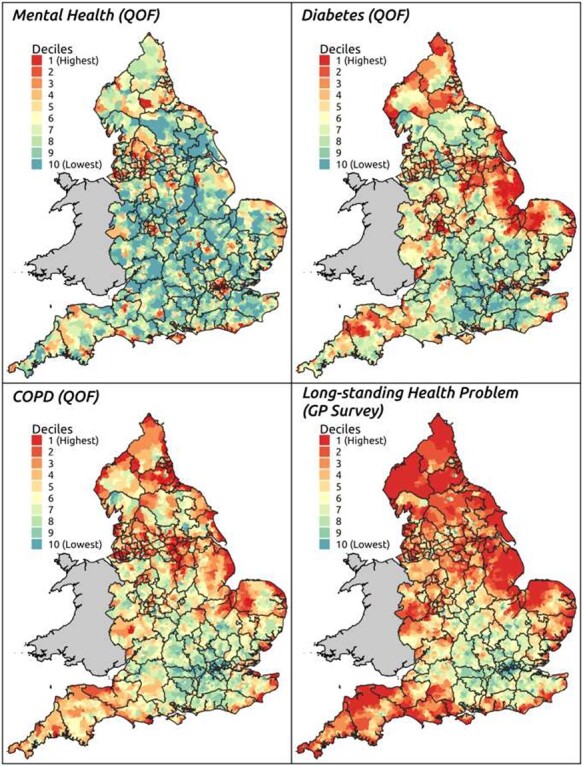
Non-communicable disease prevalence (LSOAs), 2014/15–2018/19.

Not all conditions exhibit quite such a distinct core/periphery, but coastal communities experience a higher burden of ill health across almost all conditions included in the QOF dataset, including mental health, diabetes and chronic obstructive pulmonary disease (Fig. 2). GP survey data on people reporting a longstanding health condition also indicate higher levels of ill health in coastal areas.

This unusually granular perspective has been constructed using GP-level QOF and survey data^12^ attributed to LSOAs on the basis of NHS Digital data on the LSOAs in which GP patients live.^13^ This lends a very different perspective to routinely published health, health-related and health service data, which, with few exceptions, are only made available for individual NHS provider trusts and/or large administrative areas (such as Clinical Commissioning Groups (CCGs) and Local Authorities (LAs)). This is a particular issue for coastal communities as few CCGs or LAs serve wholly, or even predominately, coastal populations. It also affects smaller towns and cities which, like coastal communities, tend to comprise but a fraction of the CCG or LA of which they are a part.^14^ As a result, evidence on the characteristics and needs such populations is subsumed within data describing the CCG/LA as a whole.

The high prevalence of non-communicable diseases (NCDs) around the coastal fringe should not be surprising as coastal communities tend to be older and more deprived than the national average. In the absence of any official definition, we have defined ‘coastal’ LSOAs as those which include or overlap a built-up area of any size which lies within 500 m of the ‘Mean High Water Mark’ coastline.^15^ On this basis, 21% of people living in coastal LSOAs are aged 65+ compared to 17.8% in non-coastal LSOAs.^16^ The 2019 Index of Multiple Deprivation,^17^ meanwhile, suggests that 16.6% of coastal residents live in one of the most deprived 10% of LSOAs in the country, whereas only 5.1% live in one of the least deprived.

Economic decline and socio-economic deprivation in coastal areas exacerbate the risk of developing NCDs, particularly at a younger age (i.e. before 65 years old). The coastal labour force tends to be relatively low skilled, low-paid and service-sector oriented. Although median weekly earnings data published by the Office for National Statistics^18^ are based on a small sample and are not considered robust, it is notable that, in 2020, nine of the 13 areas with the lowest average weekly wages were in coastal areas.

Low pay and low job security reduce access to material resources such as housing and healthy food and increase exposure to occupational hazards.^19^ Low job status with less autonomy and income insecurity are also key risk factors for chronic psychological distress,^20^^,^^21^ a risk factor for chronic inflammation and in turn the development of NCDs.^22^^,^^23^ An additional concern at present is that coastal economies, which rely disproportionately on tourism, will have been particularly hard hit by COVID.

### Children and young people’s outcomes

**Fig. 3:**
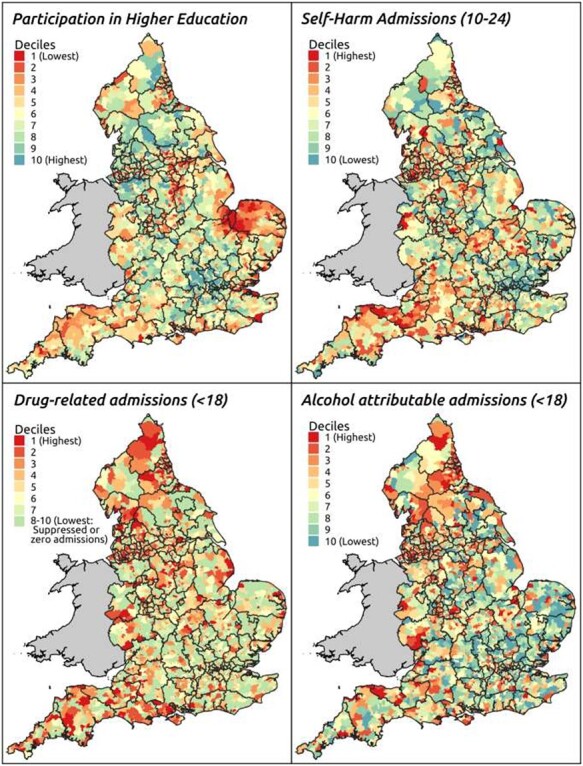
Outcomes relating to children and young people.

Although socio-economic variations in educational attainment appear to be closing, geographical variations remain pronounced. From a low base in the early 2000s, London (particularly Inner London) is the highest performing region with respect to primary and secondary school performance, progression to higher education and in closing the gap between disadvantaged and other pupils. Overall, pupils in coastal areas perform only slightly less well in their General Certificate of Secondary Education (GCSE) examinations, but achievement levels for disadvantaged pupils are considerably lower than their peers living in non-coastal locations.^24^ Geographical variation in progression to higher education is particularly pronounced (see Fig. 3).

Educational inequality is echoed in variations in health outcomes among children and young people (Fig. 3). London now has the lowest rates of hospital admissions for self-harm (aged 10–14 years), alcohol (<18 years) and substance use (<18 years). In contrast, seven out of the 10 LAs with the highest rates of hospital admissions as a result of self-harm (10–24 years) in 2018/19 were coastal.^25^ Blackpool has the highest rate of hospital admissions (aged 15–24) for substance misuse; nearly three times higher than the English average and over seven times higher than in Camden and Islington. Torbay’s rate of hospital admissions (under 18) for alcohol-specific conditions is 2.5 times the national average and 10 times higher than in Newham.

As education predicts employment, income and access to material resources as well as psychosocial well-being and health behaviours, it is arguably the single most important modifiable social determinant of health.^26^ Thus, poorer educational outcomes in coastal areas may be an early indicator of an increasing gap in health inequalities between the core and periphery.

Hundreds of studies examining a wide range of health outcomes have documented the relationship between educational outcomes and health and life expectancy^27–29^ and various mechanisms linking the two have been proposed.^30^^,^^31^ First, education leads to better-paid and more stable jobs with greater autonomy and less exposure to psychosocial stress. Second, there is growing evidence that education plays a direct role in developing psychological resilience.^32^ Resilience encompasses a range of processes that protect people from the negative effects of stress.^33–35^ It has been identified as a predictor of health in children^36^^,^^37^ and adults,^38^ possibly through protection from stress-induced immune changes.^35^^,^^39^^,^^40^ A third causal pathway linking education, resilience and health is self-efficacy, a concept that refers to an individual’s belief in their ability to exert control over their behaviour. A sense of control influences health-related behaviours and has been associated with an increased likelihood to exercise, drink moderately, not smoke and get annual health check-ups.^31^

## Factors accounting for the rise in coastal disadvantage

Why are coastal areas subject to such multiple problems? One factor is a decline in traditional industries and a failure to develop alternative sectors other than tourism. Ports have been subject to pressures from globalisation, deindustrialisation and new technologies. Overfishing and the allocation of fishing quotas to large companies has taken its toll on the fishing industry, and there has been a contraction of military facilities in waterfront locations. The loss of these industries, which have been at the heart of many coastal communities, has left new generations without secure and well-paid jobs or a clear sense of their future.

The limited range of employment opportunities available to children growing up in economically marginal coastal areas distant from large urban centres also has adverse socio-psychological consequences. London’s extraordinary educational success has been attributed to its greater ethnic diversity (children of immigrants being considered to have particularly high aspirations and ambitions^41^) and the range of opportunities available beyond the school gates.^42^ As a world city, the capital gives children exposure to a vast array of social, economic and cultural opportunities that are likely to shape their knowledge, aspirations and expectations. In contrast, children in coastal areas are often unable to see beyond the low-paid hospitality and care sectors. Indeed, the full spectrum of work opportunities may be a rather abstract concept. Poor rates of progression to higher education are significant in this respect.

Educational capital is also lower in coastal areas, with many communities having much higher than average proportions of working age adults with low or no qualifications.^43^ Families’ knowledge, information and experience of schooling^44^ all play an important role shaping children’s own aspirations and expectations.

Coastal areas have also been subject to cultural and social displacement. No longer defined by their relation to the water, traditional economic, socio-cultural and political connections have been severed for many coastal residents. There has been an exodus of younger people with higher qualifications, whereas those who stay tend to be from poorer backgrounds.^6^ Conversely, many seaside resorts have played a key role receiving out-of-area social services placements, with redundant tourist accommodation being converted into low-cost multiple occupancy dwellings to house a range of vulnerable people.^6^ People displaced from their home areas are separated from their jobs, children’s schools and vital support networks, including family networks. Such displacement is a risk factor for chronic psychological distress and in turn health inequality.

## Implications for research and policy

### Research

There are signs that coastal deprivation and associated problems are rising on the public health research agenda. The 2021 Chief Medical Officer’s report focuses on coastal health and, in 2020, the National Institute for Health Research Public Health Programme issued a call for research on reducing health inequalities in coastal towns and communities with a specific emphasis on population-level interventions. This is a very welcome development.

There is nevertheless a pressing need for a multi-disciplinary evidence-base on the nature and underlying causes of the inter-linked economic, social, environmental and service issues affecting coastal communities in England; for research that incorporates coastal stakeholder voices to ensure that an understanding of problems, interventions and solutions reflects views from the ground; and for the design and evaluation of complex interventions that incorporate sectors that have important health consequences, such as education, transport and housing.

As noted above, the lack of an official definition of coastal communities and the poor granularity of most publicly available data have rendered the coastal fringe largely invisible to detailed analysis. Access to official data for small area data should be facilitated, and the Office for National Statistics could also consider the case for developing a coastal definition.

### Policy

With respect to policy, we have noted that education is arguably the single most important modifiable social determinant of health.^26^ It is therefore concerning that school funding allocations (2020–21) remain highest in London, the region that outperforms all other English regions with respect to educational outcomes. Areas such as Tower Hamlets, Newham and Kensington and Chelsea enjoy significantly higher per capita allocations (£6947, £6192 and £6163) than Knowsley (£5383), Blackpool (£4839) and Portsmouth (£4770). Yet in these London boroughs the average GCSE performance of free school meal (FSM) pupils is higher than that of non-FSM pupils in the coastal authorities listed. Levelling up educational expenditure in areas with poor educational performance could have a transformative effect on coastal children’s life trajectories.

The adverse socio-psychological environment faced by coastal children also needs to be tackled. Within education, a vast array of extra-curriculum interventions exists.^45^ Some focus on educational outcomes; providing access to academic and study skills support, wider learning opportunities, out-of-school activities and widening participation initiatives. Others focus more generally on the healthy development of adolescents, e.g. through family support and building resilience skills which, it is argued, lead to lasting beneficial effects on a range of educational, social, economic and health outcomes.^46–48^ Community interventions involving local government, families, voluntary organisations and schools have also been advocated, including by the recent Lancet Commission on adolescence.^49^ These seek to promote life skills and positive attitudes such as self-confidence and empowerment, social and emotional skills, and good problem solving.

A growing body of research has examined the potential of mentoring to both support resilience and improve access to the academic and practical opportunities designed to improve young people’s trajectories. A mentoring relationship is generally characterised as a strong connection between an older or more experienced individual who provides guidance and support to a younger or less experienced mentee or protégé over time.^50^ Meta-analyses have found modest but significant effects of mentoring on the psychological, emotional, behavioural and educational functioning of participating youth.^51–56^ Mentoring may thus offer potential to address low levels of aspiration and ‘nothing-to-lose’ attitudes among coastal youth. A recent mapping exercise of mentoring organisations in England found that 36% undertook their work in London. With all other regions poorly represented,^55^ there is scope for providing additional support for children in disadvantaged coastal areas. The use of digital technologies, although as yet largely untested and raising questions of equity of access, may help address practical barriers of distance and the smaller pool of suitable mentors available in peripheral regions.

Although interventions such as mentoring may increase the aspirations of disadvantaged coastal children, they will not necessarily transform their opportunities. Changes to the way in which the Treasury uses gross value added (GVA) metrics to inform spending decisions (which, until recently, worked to the advantage of economically productive areas such as London and the South East) should support the case for additional infrastructural spending in the coastal periphery. Areas of potential investment include the digital economy (a sector known to have multiplier effects on employment in other sectors) and the blue and green economies. Again, there is a need for multi-disciplinary research to guide investments, perhaps following the lead of the UK Research and Innovation’s new Sustainable Management of UK Marine Resources research programme.

## Discussion

The main focus of this study has been to show that coastal communities experience a significantly higher burden of disease than their non-coastal counterparts, with particularly worrying trends in public health-related outcomes for children and young people. This has received little policy attention and attracted remarkably little research. As a perspective piece, we have only touched the surface of what are currently poorly understood patterns of disadvantage with respect to both their causes and potential solutions. We nevertheless hope that this study helps to raise awareness of the multiple and inter-linked problems facing England’s periphery and that greater priority is given by Research Councils and Government Departments to what should be seen as an important part of the levelling up agenda.

## Notes

**Sheena Asthana,** Professor

**Alex Gibson,** Research Fellow

## References

[1] MHCLG . The English Indices of Multiple Deprivation 2019. Research Report. London: MHCLG, 2019.

[2] Beatty C , Fothergill S, Gore T. Seaside Towns in the Age of Austerity: Recent Trends in Employment in Seaside Tourism in England and Wales. Sheffield: Centre for Regional Economic and Social Research, Sheffield Hallam University, 2014.

[3] Corfe S . Living on the Edge: Britain’s Coastal Communities. London: Social Market Foundation, 2017.

[4] Agarwal S , Jakes S, Page S et al. Disadvantage in English seaside resorts: a typology of deprived neighbourhoods. Tour Manag 2018;69:440–59.

[5] Corfe S . Falling off a Cliff. London: Social Market Foundation, 2019.

[6] House of Lords . The Future of Seaside Towns. Report of a Select Committee on Regenerating Seaside Towns and Communities, House of Lords Paper 320. London: House of Lords, 2019.

[7] Somerville P , Smith R, McElwee G. The dark side of the rural idyll: stories of illegal/illicit economic activity in the UK countryside. J Rural Stud 2015;39:219–28.

[8] Woodward R . ‘Deprivation’ and ‘the rural’: an investigation into contradictory discourses. J Rural Stud 1996;12:55–67.

[9] Wheeler BW , White M, Stahl-Timmins W et al. Does living by the coast improve health and wellbeing? Health Place 2012;18(5):1198–201. 22796370 10.1016/j.healthplace.2012.06.015

[10] White MP , Wheeler BW, Herbert S et al. Coastal proximity and physical activity: Is the coast an under-appreciated public health resource? Prev Med 2014;69:135–40. doi: 10.1016/j.ypmed.2014.09.016 25284259

[11] Garrett JK , Clitherow TJ, White MP et al. Coastal proximity and mental health among urban adults in England: the moderating effect of household income. Health Place 2019;59:102200. doi: 10.1016/j.healthplace.2019.102200 31582294

[12] NHS Digital (2020*)* Quality and Outcomes Framework. https://digital.nhs.uk/data-and-information/publications/statistical/quality-and-outcomes-framework-achievement-prevalence-and-exceptions-data; (23 January 2021, date last assessed). NHS (2020) *GP Patient Survey: Analysis Tool*. https://gp-patient.co.uk/analysistool. (13 January 2021, date last assessed).

[13] NHS Digital (2020) Patients Registered at a GP Practice. https://digital.nhs.uk/data-and-information/publications/statistical/patients-registered-at-a-gp-practice. (22 January 2021, date last assessed).

[14] Haynes R , Gale S. Deprivation and poor health in rural areas: inequalities hidden by averages. Health Place 2000;6(4):275–85. doi: 10.1016/s1353-8292(00)00009-5. 11027953

[15] ONS BUA/BUASD (2011). Built-up Area Sub Divisions. https://geoportal.statistics.gov.uk/datasets/built-up-area-sub-divisions-december-2011-boundaries-2. (12 November 2020, date last assessed). A number of villages and towns on tidal rivers as well as all LSOAs within the London region were excluded.

[16] Office for National Statistics (2020) Lower layer Super Output Area population estimates, Mid-2019. https://www.ons.gov.uk/peoplepopulationandcommunity/populationandmigration/populationestimates/datasets/lowersuperoutputareamidyearpopulationestimates. (10 November 2020, date last assessed).

[17] MHCLG (2019) National Statistics: English Indices of Deprivation 2019. https://www.gov.uk/government/statistics/english-indices-of-deprivation-2019. (16 January 2021, date last assessed).

[18] Office for National Statistics (2020) Earnings and Hours Worked, Place of Residence by Local Authority: ASHE Table 8. https://www.ons.gov.uk/employmentandlabourmarket/peopleinwork/earningsandworkinghours/datasets/placeofresidencebylocalauthorityashetable8. (15 January 2021, date last assessed).

[19] Bambra C . Health Divides: Where You Live Can Kill You. Bristol: Bristol University Press, 2016.

[20] Dieker AC , IJzelenberg W, Proper KI et al. The contribution of work and lifestyle factors to socioeconomic inequalities in self-rated health – a systematic review. Scand J Work Environ Health 2019;45(2):114–25. 30370911 10.5271/sjweh.3772

[21] Watson B , Osberg L. Healing and/or breaking? The mental health implications of repeated economic insecurity. Soc Sci Med 2017;188:119–27. 28750246 10.1016/j.socscimed.2017.06.042

[22] Steptoe A , Hamer M, Chida Y. The effects of acute psychological stress on circulating inflammatory factors in humans: a review and meta-analysis. Brain Behav Immun 2007;21:901–91. 17475444 10.1016/j.bbi.2007.03.011

[23] Poole L , Steptoe A. The combined association of depressive symptoms and C-reactive protein for incident disease risk up to 12 years later. Findings from the English longitudinal study of ageing (ELSA). Brain Behav Immun 2020;88:908–12. doi: 10.1016/j.bbi.2020.01.010. 31972338

[24] Department for Education (2019). Outcomes for Pupils at the End of KS4 by Geography Ad hoc Statistics. London: Department for Education. https://assets.publishing.service.gov.uk/government/uploads/system/uploads/attachment_data/file/808564/Outcomes_for_pupils_at_the_end_of_KS4_by_geography_-_ad_hoc_statistics.pdf. (18 December 2020, date last assessed).

[25] Public Health England (2020). Child and Maternal Health. https://fingertips.phe.org.uk/profile/child-health-profiles/data (15 September 2020, date last assessed). © Crown copyright 2021

[26] The Lancet Public Health . Education: a neglected social determinant of health. Lancet Public Health 2020;5(7):e361. doi: 10.1016/S2468-2667(20)30144-4. 32619534 PMC7326385

[27] Zajacova A , Lawrence EM. The relationship between education and health: reducing disparities through a contextual approach. Annu Rev Public Health 2018;39:273–89. 29328865 10.1146/annurev-publhealth-031816-044628 PMC5880718

[28] Gumà J , Solé-Auró A, Arpino B. Examining social determinants of health: the role of education, household arrangements and country groups by gender. BMC Public Health 2019;19:699. 31170953 10.1186/s12889-019-7054-0 PMC6555096

[29] Davies NM , Dickson M, Davey Smith G et al. The causal effects of education on health outcomes in the UK biobank. Nat Hum Behav 2018;2(2):117–25. 30406209 10.1038/s41562-017-0279-y PMC6217998

[30] Hahn RA , Truman BI. Education improves public health and promotes health equity. Int J Health Serv 2015;45(4):657–78. 25995305 10.1177/0020731415585986 PMC4691207

[31] Ross CE , Chia-ling W. The links between education and health. Am Sociol Rev 1995;60(5):719–45.

[32] Waxman H , Huang S. Motivation and learning environment differences between resilient and nonresilient inner-city middle school students. J Educ Res 1996;90:93–102.

[33] Windle G . What is resilience? A review and concept analysis. Rev Clin Gerontol 2011;21(2):152–69.

[34] Fletcher D , Sarkar M. Psychological resilience: a review and critique of definitions, concepts, and theory. European Psychologist 2013;18:12–23.

[35] Dantzer R , Cohen S, Russo SJ et al. Resilience and immunity. Brain Behav Immun 2018;74:28–42. doi: 10.1016/j.bbi.2018.08.010. 30102966 PMC6545920

[36] Skrove M , Romundstad P, Indredavik MS. Resilience, lifestyle and symptoms of anxiety and depression in adolescence: the young-HUNT study. Soc Psychiatry Psychiatr Epidemiol 2013;48(3):407–16. 22872359 10.1007/s00127-012-0561-2

[37] Bottolfs M , Støa EM, Reinboth MS et al. Resilience and lifestyle-related factors as predictors for health-related quality of life among early adolescents: a cross-sectional study. J Int Med Res 2020;48(2). doi: 10.1177/0300060520903656. PMC7111039 32070172

[38] Elliot AJ , Mooney CJ, Infurna FJ et al. Associations of lifetime trauma and chronic stress with C-reactive protein in adults ages 50 years and older: examining the moderating role of perceived control. Psychosom Med 2017;79:622–30 Elliot AJ, Turiano NA, et al.. Lifetime trauma, perceived control, and all-cause mortality: results from the Midlife in the United States Study*. Health Psychol* 2018;37:262–70. 28437379 10.1097/PSY.0000000000000476

[39] Sapienza JK , Masten AS. Understanding and promoting resilience in children and youth. Curr Opin Psychiatry 2011;24(4):267–73. 21546838 10.1097/YCO.0b013e32834776a8

[40] Dutcher JM , Creswell JD. The role of brain reward pathways in stress resilience and health. Neurosci Biobehav Rev 2018;95:559–67. 30477985 10.1016/j.neubiorev.2018.10.014

[41] Burgess S. (2014). Understanding the Success of London’s Schools. Bristol: Centre for Market and Public Organisation (CMPO) Working Paper, 14, 333. http://www.bristol.ac.uk/media-library/sites/cmpo/migrated/documents/wp333.pdf. (14 September 2020, date last assessed).

[42] APPG (2019). Closing the Regional Attainment Gap. London: House of Commons, All-Party Parliamentary Group on Social Mobility. https://www.suttontrust.com/our-research/appg-social-mobility-closing-the-regional-attainment-gap/. (15 September 2020, date last assessed).

[43] ONS (2020) Coastal Towns in England and Wales: October 2020: Data and Analysis on Seaside and Other Coastal Towns in England and Wales. https://www.ons.gov.uk/businessindustryandtrade/tourismindustry/articles/coastaltownsinenglandandwales/2020-10-06. (5 January 2021, date last assessed).

[44] Reay D . Miseducation: Inequality, Education and the Working Classes. London: Bristol University Press, 2017.

[45] Educational Endowment Foundation (2020). Teacher Learning Toolkit. https://educationendowmentfoundation.org.uk/evidence-summaries/teaching-learning-toolkit/https:/guidebook.eif.org.uk/. (10 December 2020, date last assessed).

[46] Masten AS . Ordinary magic. Resilience processes in development. Am Psychol 2001;56(3):227–38. 11315249 10.1037//0003-066x.56.3.227

[47] Patel V , Goodman A. Researching protective and promotive factors in mental health. Int J Epidemiol 2007;36(4):703–7. 17646185 10.1093/ije/dym147

[48] Las Hayas C , Izco-Basurko I, Fullaondo A et al. UPRIGHT, a resilience-based intervention to promote mental well-being in schools: study rationale and methodology for a European randomized controlled trial. BMC Public Health 2019;19(1):1413. 31664974 10.1186/s12889-019-7759-0 PMC6820972

[49] Patton GC , Sawyer SM, Santelli JS et al. Our future: a lancet commission on adolescent health and wellbeing. Lancet 2016;387(10036):2423–78. 27174304 10.1016/S0140-6736(16)00579-1 PMC5832967

[50] Rhodes JE , Spencer R, Keller TE et al. A model for the influence of mentoring relationships on youth development. J Community Psychol 2006;34(6):691–707.

[51] Wheeler ME , DuBois DL, Keller TE. Detailed Summary of Meta-Analysis Performed for “Review of Three Recent Randomized Trials of School-Based Mentoring: Making Sense of Mixed Findings”. Portland, OR: Portland State University, 2010.

[52] DuBois DL , Portillo N, Rhodes JE et al. How effective are mentoring programs for youth? A systematic assessment of the evidence. Psychol Sci Public Interest 2011;12(2):57–91. 26167708 10.1177/1529100611414806

[53] Tolan P , Henry D, Schoeny M et al. Mentoring interventions to affect juvenile delinquency and associated problems: a systematic review. Campbell Syst Rev 2013;9:148. doi: 10.4073/csr.2013.10.

[54] Eby LT , Allen TD, Hoffman BJ et al. An interdisciplinary meta-analysis of the potential antecedents, correlates, and consequences of protégé perceptions of mentoring. Psychol Bull 2013;139(2):441–76. 22800296 10.1037/a0029279

[55] Children’s Commissioner (2018). Forging Futures Through Mentoring A Risk Worth Pursuing? London: Office of the Children’s Commissioner. https://www.childrenscommissioner.gov.uk/wp-content/uploads/2018/04/Forging-futures-through-mentoring-CCO-April-2018-1.pdf. (6 December 2020, date last assessed).

[56] Raposa EB , Rhodes J, Stams GJJM et al. The effects of youth mentoring programs: a meta-analysis of outcome studies. J Youth Adolesc 2019;48(3):423–43. 30661211 10.1007/s10964-019-00982-8

